# Application of nanomedicine in emergency medicine; Point-of-care testing and drug delivery in twenty - first century

**DOI:** 10.1186/2008-2231-20-26

**Published:** 2012-09-10

**Authors:** Ali Pourmand, Mohammad Reza Pourmand, Justin Wang, Robert Shesser

**Affiliations:** 1 Department of Emergency Medicine, George Washington University Medical Center, 2150 Pennsylvania, Ave NW, Suite 2B, Washington, DC, 20037, USA; 2 Biotechnology Research Center, Tehran University of Medical Sciences, Tehran, Iran

## Abstract

**Abstract:**

The application of emerging nanotechnology to the practice of medicine represents a frontier of nanomedicine. Nanomedicine has been defined as a science which emphasizes the use of nanoscale tools in conjunction with background knowledge of the human body for medical diagnosis and treatment. Application of nanomedicine in EM may give EM providers the opportunity to diagnose and treat life-threatening diseases in a shorter period of time. These applications include diagnostic utilities, preventive medicine, targeted pharmacotherapy, and tissue regeneration.

## 

The concept of nanotechnology arose in the 1950s with Feynman’s vision that engineering could occur on an atomic level [1]. In the 1980s, Drexler predicted the revolutionizing of traditional manufacturing models with highly efficient and precise nanoscale processes [2]. Presently, the National Nanotechnology Initiative states that nanotechnology involves the manipulation of matter with dimensions between 1 and 100 nanometers to promote innovation across multiple disciplines. Myriad applications of nanotechnology already exist for the manufacture of consumer products, such as the incorporation of lightweight nanomaterials with dent-, scratch-, stain-, and wrinkle-resistant properties into automobiles, sports equipment, and clothing [3]. Nanotechnology also has promissing energy applications, as smaller lithium ion batteries capable of rapidly charging and discharging could increase the efficiency of mobile devices and electric automobiles [4]. Although still at a nascent stage, development of nanotechnology may lead to profound advances in manufacturing, the sciences, and medicine.

The application of emerging nanotechnology to the practice of medicine represents a frontier of nanomedicine. Nanomedicine has been defined as a science which emphasizes the use of nanoscale tools in conjunction with background knowledge of the human body for medical diagnosis and treatment [5]. By operating at molecular, intracellular, and intercellular levels, nanomedicine offers promising improvements in diagnostic utilities, preventive medicine, targeted pharmacotherapy, tissue regeneration, etc. Although largely theoretical at present, such potential improvements have significant implications for clinical practice of emergency medicine (EM).

## Nanodiagnostic medicine

Point-of-care testing: EM providers use point-of-care testing to promote rapid clinical decision-making and appropriate triage, thereby increasing emergency department (ED) throughput. These include tests for glucose, electrolytes, renal function, blood gas, pregnancy, cardiac biomarkers, and infectious diseases. Nanoscale technology allows for the development of highly sensitive point-of-care detection devices. For example, a recently developed microfluidic device with a surface plasmon resonance system demonstrated considerable sensitivity in its ability to measure a B-type natriuretic peptide concentration of 5 pg/mL [6]. Floriano et al. have suggested that using saliva-based nano-biochip tests for cardiac biomarkers in concert with electrocardiography could augment rapid diagnosis of and screening for acute myocardial infarction [7]. In addition, an assay for bacterial meningitis using quantum dots (Q-dots) could detect low levels of pathogen from a blood specimen, making possible earlier administration of antibiotics [8]. Zhao et al. have described a method of using a bioconjugated nanoparticle-based bioassay to detect a single bacterium within 20 minutes [9]. The greater sensitivity of nanomedicine point-of-care tests would therefore enable EM providers to more accurately and expeditiously diagnose and hence treat disease.

Neurologic disease: Magnetic resonance imaging (MRI) is a powerful tool for diagnosis of structural central nervous system pathology [10]. A nanoparticle-based MRI contrast agent could enhance visualization of structural or vascular abnormalities, such as in Parkinson’s disease or cerebrovascular accidents (CVAs). This method could improve the quality of noninvasive anatomic imaging to be on par with that of histological examination [11].

Cardiovascular diseases: Applications of nanomedicine in cardiovascular diseases include diagnosis and treatment of atherosclerosis and decreasing re-stenosis rates after stenting. An advantage of using a nanoscale agent is early detection of disease [12]. Although there are many applications for the diagnosis of cardiovascular disease based on nanoparticulate agents investigators in this field of medicine are starting to use nanomedicine to deliver therapeutic agents [13]. An exciting application is the nanopatterning and nanostructuring of stents makes these stents communicate with cells at nanoscale levels. This application will allow the endothelium to grow over the sent and will create an area of future research on obstructing coronary artery disease [14,15].

## Pharmacotherapy

Nanomedicine offers several strategies for improving the efficiency of pharmacotherapy. Encapsulating relatively insoluble pharmaceuticals in liposomes or micelles enhances drug solubility in the bloodstream [8,16]. These transport vehicles, exhibiting a hydrophobic core and a hydrophilic exterior, also shield toxic pharmaceuticals from an immune response, resulting in less degradation via phagocytosis [8,17]. Conjugation of pharmaceuticals to dendrimers, or artificial proteins, achieves similar results, and with the addition of site-specific surface antibodies, also allows for targeted drug delivery, resulting in decreased systemic adverse effects [16]. This strategy has demonstrated efficacy in the treatment of arthritis in rats utilizing conjugated indomethacin, with sites of inflammation targeted by a factor of 2.29 compared with unconjugated indomethacin [18]. Overall, application of nanotechnology to pharmacotherapy (e.g., analgesic, antibiotic, thrombolytic, etc.) improves drug bioavailability and potency, while reducing adverse effects.

These numerous benefits are evident when applied to the treatment of ischemic CVA in the ED. Ischemic CVA is a source of significant morbidity and mortality in the United States [19]. To restore cerebrovascular perfusion, eligible patients may receive an intravenous thrombolytic therapy, although it carries with it a risk of hemorrhagic complications. To temper this risk, Lanza et al. have advocated employing a lipid-encapsulated perfluorocarbon nanoparticle for thrombolytic delivery [20]. Its scale would limit it to the intravascular space, thereby minimizing extravasation of thrombolytic and resultant hemorrhagic complication. The efficiency of such a drug delivery mechanism would allow for a 10–100 times reduction in thrombolytic dosage, thus further decreasing the risk of hemorrhagic complication. In addition, Morrow et al. proposed utilizing perfluorocarbon nanoparticle emulsions with surface antibodies to cross-linked fibrin to target thrombi for vascular MRI, a concept which could translate to targeted thrombolytic delivery as well [17].

## Miscellaneous applications

Morrow et al. have anticipated the concept of a nanomachine, nanoscale technology which would target lesions (e.g., neurologic, cardiovascular) within a patient to modify pathophysiology at the molecular level [17]. In patients suffering from hemorrhagic CVA, prolonged arterial extravasation in the acute phase adversely affects the potential for reperfusion, as the blood brain barrier becomes more permeable via inflammatory mediators [8]. Ellis-Behnke have suggested a mechanism of lining injured cerebral arteries with a molecular coating to counter this process [8]. Haase has advocated an alternative strategy in the treatment of sepsis, the severe form of which results in nearly 600,000 ED visits annually [21,22]. In septic shock, an abnormal inflammatory response to circulating bacterial endotoxin results in greater vascular permeability and hence decreased tissue perfusion [23]. Nanotechnology could target and eliminate the cytokines which mediate this deranged systemic response, effectively functioning as a blood filtering system [21].

The future may see the synthesis of novel nanomaterials for acute management of trauma (e.g., burns, fractures) in the ED. Being biologically inert and hence less antigenic, use of these nanomaterials in tissue regeneration may eliminate the need for organic grafts and transplants, which are subject to compatibility and supply limitations [18]. In a hamster model, Ellis-Behnke has demonstrated the feasibility of using a synthetic extracellular matrix scaffold for neural regeneration of the visual tract. Similarly, carbon nanotubes show promise as a framework for bone regeneration [8]. Furthermore, these nanomaterials may be engineered to exhibit antibacterial surfaces, which may reduce the incidence of nosocomial infection [21]. These applications could be extended to experimental treatment of diabetic neuropathy and as well to eliminate colitis in rats [24,25].

Applications of nanomedicine in EM may give physicians’ opportunity to detect and treat life-threatening diseases in short period of time. These applications include diagnostic utilities, preventive medicine, targeted pharmacotherapy, and tissue regeneration. -It is worthwhile to mention that there are still some concerns regarding nanoparticles' functionality and their characteristics in vivo versus in vitro.

## Competing interests

The authors have no commercial associations or sources of support that might pose a conflict of interest.

## Authors’ contribution

All authors have made substantive contributions to the study, and all authors endorse the data and conclusions. All authors read and approved the final manuscript.
